# Changes in apolipoproteins following ingestion of a beverage delivering *β*-hydroxybutyrate: results from a randomized placebo-controlled trial

**DOI:** 10.3389/fnut.2025.1726174

**Published:** 2026-02-05

**Authors:** Yutong Liu, Wandia Kimita, Sakina H. Bharmal, Maxim S. Petrov

**Affiliations:** School of Medicine, University of Auckland, Auckland, New Zealand

**Keywords:** acute nutritional ketosis, apolipoproteins, cardiovascular risk, prediabetes, triglyceride-rich lipoproteins

## Abstract

**Background:**

Apolipoproteins play important roles in the metabolism of triglyceride-rich lipoproteins. Ketone monoester *β*-hydroxybutyrate (KEβHB) has been shown to reduce the circulating levels of remnant cholesterol and triglycerides. However, the mechanisms behind this action remain unknown.

**Aim:**

To investigate the effect of KEβHB supplementation on apolipoproteins and to study whether circulating levels of triglycerides play a role in this effect.

**Methods:**

The study was a randomized placebo-controlled trial, registered at https://www.clinicaltrials.gov/ (NCT03889210). It included 18 adults (12 men and 6 women) with prediabetes (defined as per the American Diabetes Association criteria). Following an overnight fast, participants ingested a KEβHB or a placebo beverage in a cross-over manner. Serial blood samples were collected from baseline to 150 min at intervals of 30 min. The endpoints were changes in apolipoprotein (apo) A-I, apo B, apo B-48, apo C-II, apo C-III, and apo E. Area under the curve (AUC) analyses were calculated to estimate changes in the studied apolipoproteins over time. Participants were further stratified into ‘hypertriglyceridemia’ and normal triglyceride levels subgroups.

**Results:**

Ingestion of the KEβHB beverage led to a significantly higher AUC for apo C-II (*p* = 0.023) in the overall cohort. No statistically significant differences in AUCs were found for the other studied apolipoproteins. The subgroup analysis showed significantly lower levels of apo B (and higher levels of apo C-II) after the KEβHB beverage in individuals with hypertriglyceridemia only. No significant associations were found for the other studied apolipoproteins in either subgroup.

**Conclusion:**

Exogenously induced acute ketosis resulted in a significantly elevated apo C-II compared with the placebo. Further, the levels of apo B were significantly lowered following ingestion of the KEβHB beverage only among individuals with hypertriglyceridemia. Acute nutritional ketosis may be considered as a potential approach to reduce atherogenic triglyceride-rich lipoproteins in individuals at high cardiovascular disease risk.

## 1 Introduction

Cardiovascular diseases (CVD) have emerged as the leading cause of death and disability worldwide, where 32% of all global deaths can be attributed to CVD, equivalent to 17.9 million deaths annually (1). Dyslipidemia is one of the most important risk factors for CVD (2). It is traditionally viewed that higher levels of total cholesterol and low-density lipoprotein (LDL)-cholesterol are major contributors to CVD (3). For the past 40 years, lowering LDL-cholesterol levels has been widely accepted as the primary prevention and therapeutic strategy for CVD (4). However, more recent evidence brings forth triglycerides and triglyceride-rich lipoproteins (TRL) as predictors of atherosclerosis and all-cause mortality (5, 6). Clinical focus on triglycerides and TRL as targets for reducing CVD risk has been evolving over recent years. As an example, scientific research on acute ketosis has advanced our knowledge of dyslipidemia. Acute ketosis by oral delivery of ketone βHB monoester formulation has emerged as a safe and effective means of achieving nutritional ketosis (7, 8) in both healthy individuals (9, 10) and individuals with metabolic disorders (11). A 2022 randomized controlled trial (RCT) as part of the Cross-over randomisEd Trial of β-hydroxybUtyrate in prediabeteS (CETUS) project demonstrated that acute ketosis achieved by a single dose of KEβHB significantly reduced the circulating levels of triglycerides and remnant cholesterol (i.e., the cholesterol content in TRL) (12). This opens up an opportunity for considering exogenous ketosis as a preventative or therapeutic approach to managing dyslipidemia.

Despite the marked advancements in our understanding of the beneficial effect of acute ketosis on lipid profile, the physiological mechanisms underlying its action remain poorly understood. Apolipoproteins, a known CVD risk factor, play a critical role in regulating the metabolism of lipoproteins (13). Apolipoproteins are a group of specialized proteins that reside on the surface of lipoproteins, where they carry out pivotal functions such as providing structural support, aiding the assembly of lipoprotein particles, and acting as cofactors for enzymes (14). The clinically significant apolipoproteins include the clinical significant apolipoproteins include apolipoprotein A-I (apo A-I), apolipoprotein B (apo B, includes apo B-48 and apo B-100), apolipoprotein C-II (apo C-II), apolipoprotein C-III (apo C-III), and apolipoprotein E (apo E). Specifically, as the primary apolipoprotein for TRL and their remnants, apo B is well known for its key role in atherosclerosis (15). Emerging evidence supports apo B as an accurate predictor of CVD risk—even superior to LDL cholesterol and non-high-density lipoprotein (HDL) cholesterol in both non-statin-treated (16–18) and statin-treated individuals (18, 19). Likewise, two other apolipoproteins, apo C-II and apo C-III reside on the surface of TRL [chylomicron, very low-density lipoprotein (VLDL), and intermediate-density lipoprotein (IDL)] and play important roles in TRL metabolism. Despite being within the same apolipoprotein family, apo C-II and apo C-III have opposite functions (20). Apo C-II primarily acts as a cofactor for lipoprotein lipase (LPL), which triggers LPL activity and facilitates the lipolysis of triglycerides (20). By contrast, apo C-III has an inhibitory effect on LPL, leading to reduced lipolysis of TRL particles and reduced clearance of TRL remnants (21). In addition, apo E acts as a ligand for receptor-mediated clearance of TRL from plasma (22). Defects in the apo E coding gene have been shown to be associated with elevated levels of total cholesterol, triglycerides, and increased risks of premature atherosclerosis (23). Given the involvement of apolipoproteins in the metabolism of TRL, coupled with the previously established inhibitory effect of acute ketosis on TRL, we hypothesized that apolipoproteins drive, at least in part, the observed reductions in triglycerides and remnant cholesterol levels observed following ingestion of KEβHB beverage.

The primary aim was to investigate the effect of KEβHB beverage on apolipoproteins. The secondary aim was to investigate whether circulating levels of triglycerides play a role in this effect.

## 2 Methods

### 2.1 Study design and ethics

The study was part of the CETUS project, with its primary endpoint—changes in plasma glucose levels following the consumption of a KEβHB beverage versus a placebo beverage—reported in detail elsewhere (11). The present study focused on one of the secondary endpoints—changes in lipid profile (more specifically, apolipoproteins, including apo A-I, apo B, apo B-48, apo C-II, apo C-III, and apo E). The study received ethical approval from the Health and Disability Ethics Committee, New Zealand (18/NTB/161), and was prospectively registered at https://www.clinicaltrials.gov/ (NCT03889210). Written informed consent was obtained for participation in this study. The study was conducted in accordance with the standards set by the Helsinki Declaration.

### 2.2 Study participants and randomization

Eligible for participation were men and women of 18 years and above who met the American Diabetes Association criteria for prediabetes: glycated hemoglobin (HbA1c) between 5.7 and 6.4% (39–47 mmol/mol) and/or fasting plasma glucose (FPG) between 100 and 125 mg/dL (5.6–6.9 mmol/L) (24). Exclusion criteria were a diagnosis of diabetes prior to participation in the study; use of anti-diabetic or lipid-modifying medications or corticosteroids; a history of ketogenic diets or taking ketone supplements; participation in competitive sports or intensive physical training programs regularly; history of bariatric surgery or gastrointestinal surgery; malignancy; pregnancy or post-partum. An online number generator was used to randomly assign participants to either a KEβHB beverage or a placebo beverage with an allocation ratio of 1:1 and a block size of 4. Neither the participants nor the research nurse who collected blood samples was aware of the allocation sequence.

### 2.3 Study protocol

The study took place at the University of Auckland (Auckland, New Zealand). Participants visited the clinic on two occasions. They were required to fast for over 8–10 h, and to refrain from exercise and alcohol for at least 24 h prior to each clinic visit. Participants were asked to record their food intake 24 h before their first clinic visit, and they were requested to replicate their dietary intake 24 h before their second clinic visit (25–27).

During the first clinic visit, a venous catheter with an integrated stopcock was inserted for serial blood collection, and fasting blood samples were obtained. Immediately following the collection of fasting blood samples, participants were asked to consume a single dose of KE*β*HB beverage (≤123 kcal in energy) or a placebo beverage (energy content not measured) (28, 29). The KEβHB beverage was prepared using a commercially available ketone *β*-hydroxybutyrate monoester supplement (R-3-hydroxybutryl-1,3-hydroxybutyrate, *∆* G^®^; ∆ TS Ltd), flavored stevia (SweetLeaf Sweetener^®^), and water, and it was individually lean-weight-dosed at 1.9 kcal/kg (equivalent to 1.05 mL/kg, 395 mg/kg), providing 4.4 mmol/kg of D-*β-*hydroxybutyrate. The placebo beverage consisted of water, favored stevia (SweetLeaf Sweetener^®^), malic acid (BioTrace^®^), and arrowroot (MC Kenzie’s^®^). Both beverages were attempted to match in color and viscosity. Serial blood samples were sequentially collected at 30, 60, 90, 120, and 150 min following the consumption of beverages. Participants were required to remain sedentary throughout the study period (30).

The second clinic visit took place after a mean [± standard deviation (SD)] washout period of 8.2 ± 2.9 days. The protocol mentioned above was repeated for participants’ second clinic visit, except that the alternate beverage was given.

### 2.4 Laboratory measurements

Lithium heparin tubes and EDTA tubes were used to collect serial blood samples. Fresh, never frozen blood samples were sent to LabPlus—an accredited tertiary medical laboratory located at Auckland City Hospital (New Zealand)—for the analysis of total cholesterol, HDL cholesterol, triglycerides, plasma glucose, and HbA1c. *β*-hydroxybutyrate levels were measured using a handheld ketone meter and FreeStyle Optium *β*-ketone test strip (Abbott Laboratories) on whole blood. Blood samples used to measure levels of apolipoproteins were centrifuged at 4 °C and at a speed of 4,000 g for 5 min immediately after collection. The serum was then aliquoted, transferred, and stored at −80 °C until analysis. Apolipoproteins (apo A-I, apo B, apo C-II, apo C-III, apo E) were measured using the MILLIPLEX^®^ MAP Human Apolipoprotein Magnetic Bead Panel (Cat # HAP0-8062; Millipore, USA). Apo B-48 was measured using Human Apolipoprotein B-48 (APOB-48) ELISA Kit (Cat # abx574262; Abbexa, Cambridge, UK). The immunoreactive mass of plasma LPL was measured using an enzyme-linked immunosorbent assay (ELISA) analysis kit (Cloud-Clone Corp., Houston, TX, USA). All assays were performed as per the manufacturers’ instructions.

### 2.5 Statistical analysis

SPSS software version 28.0 (IBM Corporation) and Prism software version 9 (GraphPad) were used for statistical analyses. Data were presented as mean ± SD if they were normally distributed variables as evidenced by the Shapiro–Wilk normality test, or otherwise presented as median and interquartile range (31). For all analyses, *p*-values < 0.05 were accepted as being statistically significant. The statistical analyses were conducted as follows:

First, data were log-transformed prior to analyses. The trapezoidal method was used to calculate the total area under the curve (AUC) for apolipoproteins from baseline to 150 min. Total AUCs were presented as mean ± standard error of the mean (SEM), and the paired sample *t*-test was used to compare the total AUCs of apolipoproteins after the KEβHB beverage and the placebo. Cohen’s d was used to present the magnitude of difference in the total AUCs between the two groups.

Second, repeated measures two-way analysis of variance (ANOVA) with Geisser–Greenhouse correction was used to assess changes in apolipoproteins over six time points (time effect) between the KEβHB beverage and the placebo (treatment effect), as well as the interaction between the time and treatments (time × treatment interaction effect) (32). *Post-hoc* multiple comparisons with Sidak correction between groups (KEβHB beverage versus placebo beverage) at each time point (0, 30, 60, 90, 120, 150 min) were conducted only when a significant interaction effect was found.

Third, the participants were stratified into the ‘hypertriglyceridemia’ and ‘normal triglycerides’ subgroups based on the median values of their circulating triglyceride levels, which were calculated by averaging the triglyceride levels measured at baseline (0 min) from both study visits (before consuming the KEβHB beverage and the placebo beverage). The statistical analysis was analogous to the main analysis described above.

## 3 Result

### 3.1 Characteristics of study participants

**Figure 1:**
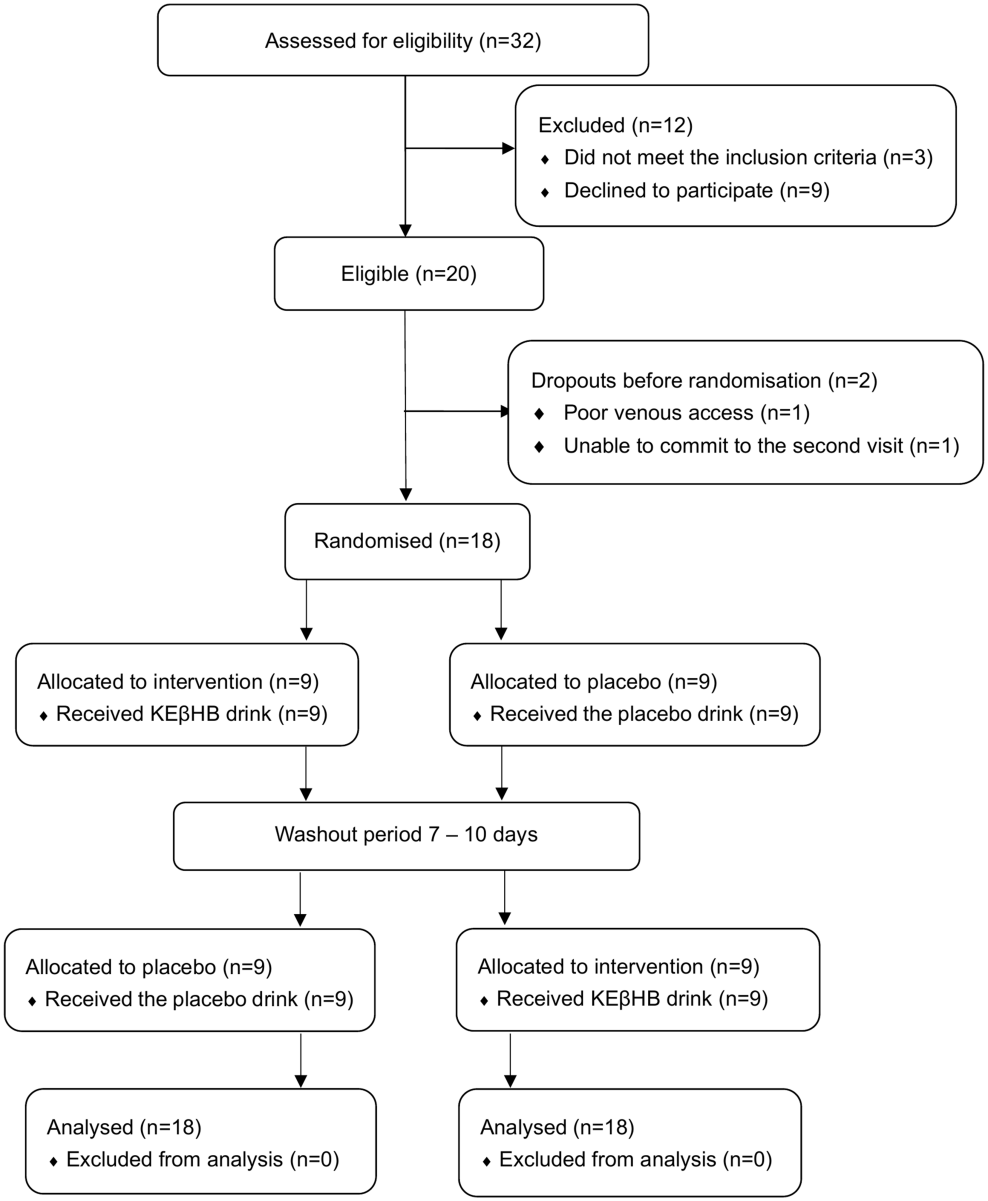
Consolidated standards of reporting trials (CONSORT) diagram.

A total of 18 participants (6 women and 12 men) met the eligibility criteria and completed the study (Figure 1). None of the study participants were deficient in LPL, with a median LPL concentration of 205.33 ng/mL at baseline in the overall cohort, ranging from 57.0 to 842.76 ng/mL. Other characteristics of the study participants are presented in Table 1.

**Table 1:** Participant characteristics at baseline.

Characteristic	*N* = 18^a^
Sex (%)	67% M (*n* = 12); 32% F (*n* = 6)
Age (years)	58.0 (45.50, 67.50)
Systolic blood pressure (mmHg)	135 ± 27
Diastolic blood pressure (mmHg)	88 ± 13
Body mass index (kg/m^2^)	28.4 ± 5.9
Triglyceride	168.30 (110.70, 199.30)
Total cholesterol (mg/dL)	195.30 (163.40, 216.60)
HDL cholesterol (mg/dL)	46.40 (41.57, 52.34)
LDL cholesterol (mg/dL)	121.70 (88.32, 139.80)
Hepatic lipase (ng/mL)	9264.18 (7725.58, 11770.58)
Apo A-I (mg/dL)	205.89 (95.26, 438.83)
Apo B (mg/dL)	60.91 (47.15, 174.95)
Apo B-48 (mg/dL)	14.50 (12.76, 16.46)
Apo C-II (mg/dL)	1.55 (0.92, 10.26)
Apo C-III (mg/dL)	3.81 (2.84, 44.34)
Apo E (mg/dL)	6.68 (5.17, 8.01)

a Values are presented as mean ± standard deviation or median and interquartile range (unless indicated otherwise).

Apo, apolipoprotein; HDL, high-density lipoprotein; LDL, low-density lipoprotein.

### 3.2 Changes in blood βHB concentrations

Significant elevation in the level of βHB was achieved from baseline (0.18 ± 0.07 mmol/L) to 30 min (3.47 ± 0.92 mmol/L, *p* < 0.001) following the consumption of KEβHB beverage, and it remained elevated at 60 min (2.88 ± 0.46 mmol/L, *p* < 0.001), 90 min (2.17 ± 0.49 mmol/L, *p* < 0.001), 120 min (1.24 ± 0.41 mmol/L, *p* < 0.001), and 150 min (0.77 ± 0.33 mmol/L, *p* < 0.001). In contrast, no significant changes in the levels of βHB from baseline (0.17 ± 0.07 mmol/L) were observed following the placebo beverage at any time points.

### 3.3 Effect of the KEβHB beverage versus placebo on apolipoproteins

#### 3.3.1 Apo A-I

**Figure 2:**
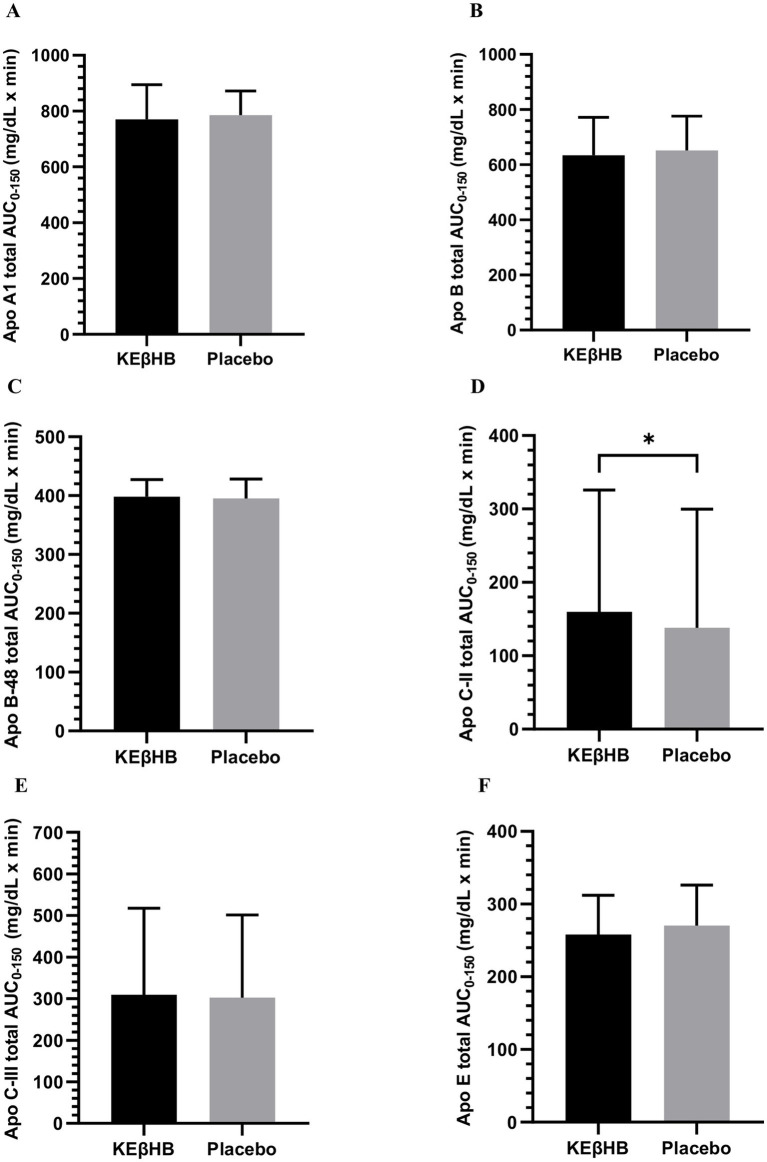
Total AUCs of **(A)** plasma concentration of Apolipoprotein A-I, **(B)** Apolipoprotein B, **(C)** Apolipoprotein B-48, **(D)** Apolipoprotein C-II, **(E)** Apolipoprotein C-III, **(F)** Apolipoprotein E in individuals with prediabetes after the KEβHB versus placebo. Total area under the curve (AUC _0–150_) in both the KEβHB and placebo beverages are presented as mean ± standard error of mean in **A–F**. Apo, apolipoprotein; AUC, area under the curve; KEβHB, ketone monoester (*β*-hydroxybutyrate). *represents statistically significant differences between groups as per the paired sample *t*-test.

Changes in apo A-I at individual time points are presented in Table 2. The total AUC_0-150_ for apo A-I following the consumption of KEβHB was 770.37 ± 29.24 mg/dL × min versus 785.31 ± 20.46 mg/dL × min following the placebo (*p* = 0.410; *d* = 0.14) (Figure 2A). There were no significant interactions of time × intervention (*p* = 0.848), main treatment effect (*p* = 0.678), or main time effect (*p* = 0.654) for apo A-I.

**Table 2:** Effect of the KEβHB versus placebo on apolipoproteins at individual time points.

Parameter^a^	Group/significance	Time point (min)					
		0	30	60	90	120	150
Apo A-I (mg/dL)	KEβHB (*n* = 18) Placebo (*n* = 18) *Δ* (KEβHB-placebo) *p-*value^b^	220.20 (71.96, 572.82) 204.94 (93.49, 421.83) 25.48 (−59.53, 164.69) 0.679	274.91 (97.51, 435.08) 177.57 (131.17, 350.31) −4.35 (−124.73, 185.30) 0.965	138.90 (77.36, 245.14) 191.47 (120.54, 355.98) −10.73 (−176.21, 100.43) 0.351	149.03 (86.55, 391.54) 221.89 (99.72, 363.09) 9.49 (−147.12, 86.04) 0.613	187.38 (121.90, 321.34) 185.87 (87.65, 330.64) 9.35 (−112.60, 103.88) 0.763	131.27 (63.84, 426.88) 201.70 (124.51, 351.86) −23.91 (−155.81, 103.98) 0.306
Apo B (mg/dL)	KEβHB (*n* = 18) Placebo (*n* = 18) *Δ* (KEβHB-placebo) *p-*value	66.48 (43.84, 159.32) 59.30 (43.89, 156.78) −7.66 (−21.11, 8.76) 0.851	64.43 (47.64, 175.35) 59.44 (43.0, 172.48) 0.36 (−11.32, 25.6) 0.714	49.63 (27.13, 154.77) 54.35 (38.81, 161.11) −3.83 (−39.61, 29.98) 0.281	59.01 (28.20, 177.99) 55.79 (43.34, 225.50) −14.39 (−44.96, 10.04) 0.270	68.86 (28.59, 135.21) 57.60 (42.79, 170.93) −7.63 (−40.54, 27.3) 0.240	59.11 (25.97, 173.16) 49.80 (39.24, 1543.98) 5.76 (−11.42, 20.94) 0.937
Apo B-48 (mg/dL)	KEβHB (*n* = 18) Placebo (*n* = 18) Δ (KEβHB-placebo) *p-*value	13.78 (12.62, 16.76) 14.01 (12.66, 16.40) 0.31 (−0.15, 1.10) 0.551	14.60 (12.54, 16.09) 13.43 (12.59, 16.62) 0.15 (−0.25, 2.11) 0.236	13.66 (12.57, 16.65) 13.82 (12.55, 17.79) −0.01 (−0.49, 0.95) 0.916	14.52 (12.92, 16.88) 13.99 (12.56, 17.40) 0.29 (−1.49, 1.35) 0.960	15.15 (12.54, 17.27) 14.41 (12.56, 15.93) 0.07 (−0.37, 1.34) 0.391	13.84 (12.29, 16.42) 13.83 (12.58, 16.91) 0.39 (−0.79, 1.36) 0.689
Apo C-II (mg/dL)	KEβHB (*n* = 18) Placebo (*n* = 18) *Δ* (KEβHB-placebo) *p-*value	1.52 (0.95, 9.38) 1.58 (0.98, 10.75) −0.14 (−0.48, 0.47) 0.798	1.39 (0.84, 10.69) 1.42 (0.98, 8.60) −0.05 (−0.43, 0.34) 0.930	1.47 (0.70, 9.89) 1.25 (1.07, 8.21) −0.16 (−0.66, 0.67) 0.764	1.40 (0.79, 8.84) 1.26 (0.97, 7.97) −0.07 (−0.96, 0.38) 0.536	1.29 (1.0, 8.64) 1.27 (0.86, 7.28) 0.14 (−0.35, 0.77) 0.202	1.33 (0.85, 9.27) 1.24 (0.89, 7.28) −0.02 (−0.51, 0.47) 0.472
Apo C-III (mg/dL)	KEβHB (*n* = 18) Placebo (*n* = 18) Δ (KEβHB-placebo) *p-*value	3.68 (2.78, 37.78) 3.57 (2.93, 49.70) −0.27 (−0.87, 0.34) 0.448	3.55 (2.71, 48.47) 3.50 (2.90, 45.15) −0.04 (−0.53, 1.74) 0.987	3.66 (2.55, 49.55) 3.43 (3.09, 35.85) −0.11 (−0.69, 1.78) 0.449	3.50 (2.41, 46.41) 3.41 (3.15, 38.21) −0.07 (−1.08, 2.54) 0.771	3.31 (2.79, 47.71) 3.83 (2.79, 49.19) 0.02 (−2.52, 1.38) 0.644	3.29 (2.75, 52.43) 3.16 (2.75, 38.65) −0.01 (−1.46, 0.84) 0.390
Apo E (mg/dL)	KEβHB (*n* = 18) Placebo (*n* = 18) *Δ* (KEβHB-placebo) *p-*value	6.94 (5.29, 8.23) 6.16 (4.85, 7.57) 0.07 (−0.76, 1.47) 0.557	5.65 (4.83, 7.55) 5.68 (4.75, 8.07) −0.77 (−1.59, 0.57) 0.184	4.92 (3.86, 7.45) 5.73 (4.89, 7.45) −0.17 (−1.06, 0.71) 0.349	5.66 (4.10, 7.61) 6.17 (4.74, 7.92) −0.57 (−2.34, 1.17) 0.158	5.62 (4.72, 7.44) 5.97 (4.97, 6.90) 0.40 (−1.06, 1.66) 0.651	5.68 (4.03, 7.86) 5.90 (5.16, 7.23) < 0.01 (−0.75, 1.73) 0.812

a Values of the studied parameters are presented as median and interquartile range, and their changes are presented as median difference and interquartile range.

b *p*-values were obtained from paired *t*-test; variables were log-transformed for the paired *t*-test.

Apo, apolipoprotein; KEβHB, ketone monoester (*β*-hydroxybutyrate).

#### 3.3.2 Apo B

Changes in apo B at individual time points are presented in Table 2. The total AUC_0–150_ for apo B following the consumption of KEβHB was 634.79 ± 32.43 mg/dL × min versus 651.92 ± 29.31 mg/dL × min following the placebo (*p* = 0.258; *d* = 0.13) (Figure 2B). There were no significant interactions of time × intervention (*p* = 0.789), main treatment effect (*p* = 0.747), or main time effect (*p* = 0.253) for apo B.

#### 3.3.3 Apo B-48

Changes in apo B-48 at individual time points are presented in Table 2. The total AUC_0–150_ for apo B-48 following the consumption of KEβHB was 398.23 ± 6.80 mg/dL × min versus 395.25 ± 7.75 mg/dL × min following the placebo (*p* = 0.555; *d* = 0.10) (Figure 2C). There were no significant interactions of time × intervention (*p* = 0.853), main treatment effect (*p* = 0.775), or main time effect (*p* = 0.580) for apo B-48.

#### 3.3.4 Apo C-II

Changes in apo C-II at individual time points are presented in Table 2. The total AUC_0–150_ for apo C-II following the consumption of KEβHB was 159.88 ± 39.13 mg/dL × min versus 138.08 ± 38.12 mg/dL × min following the placebo (*p* = 0.023; *d* = 0.13) (Figure 2D). There were no significant interactions of time × intervention (*p* = 0.410), main treatment effect (*p* = 0.944), or main time effect (*p* = 0.945) for apo C-II.

#### 3.3.5 Apo C-III

Changes in apo C-III at individual time points are presented in Table 2. The total AUC_0–150_ for apo C-III following the consumption of KEβHB was 309.72 ± 49.03 mg/dL × min versus 302.33 ± 46.96 mg/dL × min following the placebo (*p* = 0.597; *d* = 0.04) (Figure 2E). There were no significant interactions of time × intervention (*p* = 0.778), main treatment effect (*p* = 0.920), or main time effect (*p* = 0.577) for apo C-III.

#### 3.3.6 Apo E

Changes in apo E at individual time points are presented in Table 2. The total AUC_0–150_ for apo E following the consumption of KEβHB was 258.14 ± 12.77 mg/dL × min versus 270.37 ± 13.15 mg/dL × min following the placebo (*p* = 0.172; *d* = 0.22) (Figure 2F). There were no significant interactions of time × intervention (*p* = 0.731), main treatment effect (*p* = 0.545), or main time effect (*p* = 0.095) for apo E.

### 3.4 Role of levels of triglycerides

#### 3.4.1 Hypertriglyceridemia

The total AUCs for apo A-I (*p* = 0.334), apo B-48 (*p* = 0.216), apo C-III (*p* = 0.126), and apo E (*p* = 0.077) were not significantly different between the KEβHB and placebo in individuals with hypertriglyceridemia (Supplementary Table 1). However, there were significant differences in the total AUCs for apo B (*p* = 0.047) and apo C-II (*p* = 0.031) between the KEβHB and placebo in individuals with hypertriglyceridemia (Supplementary Figures 2B,D).

The KEβHB did not have a significant treatment effect on apo A-I (*p* = 0.665), apo B (*p* = 0.609), apo B-48 (*p* = 0.702), apo C-II (*p* = 0.960), apo C-III (*p* = 0.949), and apo E (*p* = 0.601), or an interaction effect on apo A-I (*p* = 0.312), apo B (*p* = 0.10), apo B-48 (*p* = 0.121), apo C-III (*p* = 0.090), and apo E (*p* = 0.195), or a main time effect on apo A-I (*p* = 0.861), apo B (*p* = 0.314), apo B-48 (*p* = 0.607), apo C-II (*p* = 0.736), apo C-III (*p* = 0.640), and apo E (*p* = 0.133) in individuals with hypertriglyceridemia. However, there is a significant interaction effect on apo C-II (*p* = 0.014) in individuals with hypertriglyceridemia.

No significant differences in apo C-II were found when comparing the KEβHB and placebo beverages at baseline (*p* = 0.769), 30 min (*p* = 0.221), 90 min (*p* = 0.160), 120 min (*p* = 0.126), and 150 min (*p* = 0.828) using post-doc multiple comparison tests. However, there was a significant difference in apo C-II between beverages at 60 min (*p* = 0.021).

#### 3.4.2 Normal triglyceride levels

The total AUCs for apo A-I (*p* = 0.834), apo B (*p* = 0.990), apo B-48 (*p* = 0.989), apo C-II (*p* = 0.161), apo C-II (*p* = 0.437), and apo E (*p* = 0.754) were not significantly different between the KEβHB and placebo in individuals with normal serum triglyceride levels (Supplementary Table 1).

The KEβHB did not have a significant treatment effect on apo A-I (*p* = 0.878), apo B (*p* = 0.972), apo B-48 (*p* = 0.991), apo C-II (*p* = 0.864), apo C-III (*p* = 0.825), and apo E (*p* = 0.789), or an interaction effect on apo B (*p* = 0.717), apo B-48 (*p* = 0.369), apo C-II (*p* = 0.943), apo C-III (*p* = 0.648), and apo E (*p* = 0.910), or a main time effect on apo A-I (*p* = 0.809), apo B (*p* = 0.486), apo B-48 (*p* = 0.390), apo C-II (*p* = 0.855), apo C-III (*p* = 0.445), and apo E (*p* = 0.504) in individuals with normal serum triglyceride levels. However, there was a significant interaction effect on apo A-I (*p* = 0.037) in individuals with normal serum triglyceride levels. No significant differences in apo A-I were found when comparing the KEβHB and placebo beverages at baseline (*p* = 0.268), 30 min (*p* = 0.064), 60 min (*p* = 0.576), 90 min (*p* = 0.766), 120 min (*p* = 0.229), and 150 min (*p* = 0.210) using post-doc multiple comparison tests (Table 3).

**Table 3:** Overall effect of the KEβHB versus placebo on apolipoproteins.

AUCs^a^	KEβHB (*n* = 18)	Placebo (*n* = 18)	Mean difference (95% CI)	*d* (effect size)	*p*-value
Apo A-I (mg/dL × min)	770.37 ± 29.24	785.31 ± 20.46	−14.94 (−52.24, 22.36)	0.14	0.410
Apo B (mg/dL × min)	634.79 ± 32.43	651.92 ± 29.31	−17.13 (−48.02, 13.76)	0.13	0.258
Apo B-48 (mg/dL × min)	398.23 ± 6.80	395.25 ± 7.75	2.98 (−7.47, 13.44)	0.10	0.555
Apo C-II (mg/dL × min)	159.88 ± 39.13	138.08 ± 38.12	21.80 (3.35, 40.25)	0.13	0.023^*^
Apo C-III (mg/dL × min)	309.72 ± 49.03	302.33 ± 46.96	7.39 (−21.56, 36.34)	0.04	0.597
Apo E (mg/dL × min)	258.14 ± 12.77	270.37 ± 13.15	−12.23 (−30.30, 5.85)	0.22	0.172

a AUCs were the total AUCs of log-transformed variables that were calculated from 0 to 150 min. Mean difference (95% CI), *d* values, and *p-*values were obtained from paired *t*-test.

Values are presented as mean ± standard error of mean (SEM).

Apo, apolipoprotein; AUC, area under the curve; CI, confidence interval; KEβHB, ketone monoester (*β*-hydroxybutyrate).

*indicates statistically significant difference between the KEβHB and placebo drinks.

## 4 Discussion

The present study was a logical continuation of the earlier CETUS study investigating the effect of acute ketosis on the standard lipid panel in humans (12). In that study, we reported significant reductions in TRL (as approximated by changes in remnant cholesterol and triglycerides) following ingestion of the KEβHB beverage in comparison with the placebo, and the effects were more evident in individuals with high habitual saturated fat intake (12). As part of the CETUS project, the present study advanced our knowledge further and reported a significantly higher level of apo C-II, but not apo C-III, following ingestion of the KEβHB beverage compared with the placebo. Moreover, the plasma level of apo B, but not apo B-48, was significantly reduced following ingestion of the KEβHB beverage among individuals with hypertriglyceridemia. Significant changes in apolipoproteins over just 150 min following ingestion of the beverage delivering *β*-hydroxybutyrate is a noteworthy finding, as acute responses of apolipoproteins are not generally expected due to their longer half-lives compared to smaller metabolites and hormones.

Owing to their high triglyceride content, TRL have highly atherogenic properties (33). Evidence from genetic and epidemiological studies supported an elevated postprandial chylomicron production as a causal risk factor for low-grade inflammation, atherosclerosis, CVD (34), and all-cause mortality (35). Further, it was suggested that VLDL promoted the formation of macrophage-derived foam cells—a crucial step in the development of atherosclerosis (36). In view of their highly atherogenic nature, the lipolysis (or clearance) of TRL inevitably becomes a key step in the control of circulating triglyceride levels and the resultant CVD risks (37). LPL is the chief player in triglyceride lipolysis of TRL. Following synthesis, it is translocated to the luminal surface of the nearby capillary endothelial cells, where it becomes responsible for the hydrolysis of triglycerides on circulating lipoproteins (38). Therefore, as the rate-limiting step for the hydrolysis and clearance of TRL (38), it is likely that any factors influencing LPL activity will have a significant impact on the circulating levels of TRL. LPL activity is controlled by several factors, both positively and negatively (39). For example, the two members of the apo C family—apo C-II and apo C-III—exert the opposite effects on LPL. Apo C-II plays a critical role in TRL metabolism as a cofactor for LPL and a stimulator for its activity. VLDL particles with apo C-II attached on the surface are secreted into the systemic circulation from the liver, where they subsequently bind to LPL on endothelial cells and generate remnants (40). Evidence from cross-linking studies suggested that the C-terminus of apo C-II was linked to the amino acid residues near the lid region of LPL molecules, promoting the displacement of the lipid and the entry of triglyceride into the active enzymatic site (40). Additionally, as the surface pressure of TRL molecules gradually accumulates from lipolysis, it becomes difficult for LPL to attach to the TRL surface, whereas apo C-II acts as a bridge, allowing LPL to retain and remain active (41). Evidence from genetic studies demonstrated an 11-fold increase in native LPL activity in the presence of apo C-II (42). Individuals with defects in the apo C-II coding gene (APOC2) are often diagnosed with hypertriglyceridemia and chylomicronemia syndrome, as the APOC2 mutation results in the poor binding of apo C-II to LPL (40). D6PV, a peptide simulating the function of apo C-II, was recently targeted as a pharmaceutical means of lowering triglycerides, where a nearly 80% reduction in triglycerides was reported (43). AUC_0–150_ was significantly reduced following KEβHB ingestion, likely reflecting rapid redistribution of pre-existing apo C-II to activate LPL during ketone-driven triglyceride clearance. Taken together with our findings on acute ketosis and its effects on lipid profile, it is conceivable that the significant reductions in TRL (as approximated by triglyceride and remnant cholesterol) during acute ketosis are, at least in part, due to the actions of apo C-II.

The present study also investigated, for the first time, the role of high triglyceride levels in the relationship between acute ketosis and TRL. We found significant reductions in the levels of apo B following ingestion of KEβHB beverages in individuals with high circulating triglyceride levels (above cohort median) at baseline. As the primary apolipoprotein residing on the surface of LDL, TRL, and their remnants, apo B is known for its key role in the initiation of atherosclerosis (15). Previous models suggested that due to the interaction of the positively charged amino acid residues on apo B and the negatively charged sulphate groups of subendothelial proteoglycans, apo B-containing lipoproteins, such as LDL, readily enter into and stay trapped in the arterial intimal space after penetrating the protective endothelial layer of the arterial wall (44). It is even more so for TRL (namely IDLs, chylomicron remnants, and VLDL) as they are larger in size (35). Meanwhile, the retained lipoproteins in the extracellular matrix became more exposed to enzymatic modification, further promoting their accumulation and aggregation. The aggregated apo B lipoproteins are ultimately taken up by macrophages, which initiates the process of transformation into foam cells (45). This conversion of macrophages to foam cells stimulates the synthesis of lipoprotein-binding proteoglycans with greater affinity, further reinforcing this vicious cycle of retention and aggregation (46). Moreover, the aggregated apo B lipoproteins enhance the release of biological byproducts that attract macrophages and smooth muscle cells to the developing lesion while discouraging them from leaving the arterial intima—a powerful combination that promotes the inflammatory response and the progression of plaque (45). Naturally, reductions in apo B will result in lower risks of atherosclerosis and CVD, as was suggested by meta-analyses (47, 48). Notably, in both the previous and present studies from the CETUS project, we observed a similar pattern of changes following ingestion of the KEβHB beverage—acute ketosis exerted greater health benefits or more pronounced improvements in lipid profile among individuals with high CVD risks (as evidenced by subgroups of high baseline triglyceride levels and greater habitual saturated fat intake). This finding strengthened the evidence base for considering exogenous ketone supplementation as a protective/therapeutic option that targets TRL in individuals with high CVD risk.

The metabolism and transportation of triglycerides and TRL involve both endogenous and exogenous pathways (47). The endogenous pathway primarily involves the liver. As the liver constantly synthesises triglycerides from carbohydrates and free fatty acids as substrates, these triglycerides are subsequently released into circulation after being packed within the core of VLDL particles (49). In this process, apo B-100 plays an essential role in the formation and trafficking of VLDL (49). In the state of lipid abundance, apo B-100 is translocated into the rough endoplasmic reticulum of hepatocytes, where primordial lipoproteins known as pre-VLDLs are produced by linking triglycerides to apo B-100. Pre-VLDL is subsequently lipidated into mature VLDL in the smooth endoplasmic reticulum and released into circulation to transport triglycerides to extra-hepatic organs (15). On the other hand, the exogenous pathway begins with the intestinal absorption of dietary triglycerides. Following absorption, triglycerides are re-esterified in the mucosal cells of the intestine, and are subsequently assembled into lipoprotein particles (with other components such as phospholipids and unesterified cholesterol) known as chylomicron (50). Chylomicron serves an important function of transporting dietary fat from the intestine to the liver (34). It uses a different apolipoprotein—apo B-48 for its assembly and secretion. Apo B-48 is known for being a specific marker for intestinal chylomicron (51). Evidence suggested that human subjects with CVD displayed a pattern of elevated plasma apo B-48 levels, impaired clearance of apo B-48-containing chylomicrons, and higher triglyceride levels than their healthy counterparts (52, 53). Taking into account the fact that, in the present study, significant differences in apo B were found between the study conditions in the subgroup analysis—a relationship that was not significant in apo B-48, it is conceivable that the changes in TRL due to acute ketosis observed in the previous study were not of gastrointestinal origin. These findings suggest that, first, the KEβHB beverage elicits its effect on TRL by acting on the liver (i.e., the endogenous pathway) and not the intestine (i.e., the exogenous pathway). Second, VLDL and chylomicrons are both cleared from circulation using a similar saturable mechanism in a competitive manner (54). Therefore, the reductions in TRL following the ingestion of the KEβHB beverage involve changes other than chylomicrons, meaning they were likely to be VLDL or IDL.

The limitations of the study need to be acknowledged. First, although we had demonstrated statistically significant changes in apo C-II and apo B, the CETUS project was not powered to investigate differences in other apolipoproteins. Therefore, we cannot rule out the presence of a type II error regarding these apolipoproteins. Second, despite the fact that we have measured the LPL levels for all participants at baseline, and none had low circulating levels of LPL, no molecular genetic testing or LPL activity assay was performed to rule out the possibility of familial LPL deficiency (55). However, the clinical presentation for LPL deficiency is elevated plasma triglyceride levels of more than 1,000 mg/dL (56), which was not the case for our study participants. Third, although we have examined and presented the changes in the levels of apo B and apo B-48 following ingestion of the KEβHB beverage, we did not directly measure the level of apo B-100. However, it has been well-established that apo B is encoded by the APOB gene, and it only exists in two forms: the full-length apo B-100 and the truncated form apo B-48 (57). Last, due to the small sample size, we did not use the standard published guidelines to identify individuals with hypertriglyceridemia (58). Rather, we used the median triglyceride values presented in our cohort of participants to identify those with low and high triglyceride levels. More research is needed in individuals with clinically defined hypertriglyceridemia.

## 5 Conclusion

As the first RCT investigating the role of apolipoproteins in the setting of exogenously induced acute ketosis in humans, the present study provides novel in-depth mechanistic insights into the impact of KE*β*HB ingestion on serum lipid profile, an action that appears to be mediated primarily through apo C-II. This goes some way to strengthen the evidential base for consideration of acute nutritional ketosis as a means of reducing atherogenic TRL in individuals with high CVD risk.

## Data Availability

The raw data supporting the conclusions of this article will be made available by the authors, without undue reservation.
