# Supplementary material for: Changes in apolipoproteins following ingestion of a beverage delivering β-hydroxybutyrate: results from a randomized placebo-controlled trial
Source: Front Nutr. 2026 Feb 5;12:1726174. doi: 10.3389/fnut.2025.1726174 (PMC12916354; doi:10.3389/fnut.2025.1726174)
Supplement: Supplementary file 1 [file Data_Sheet_1.pdf]

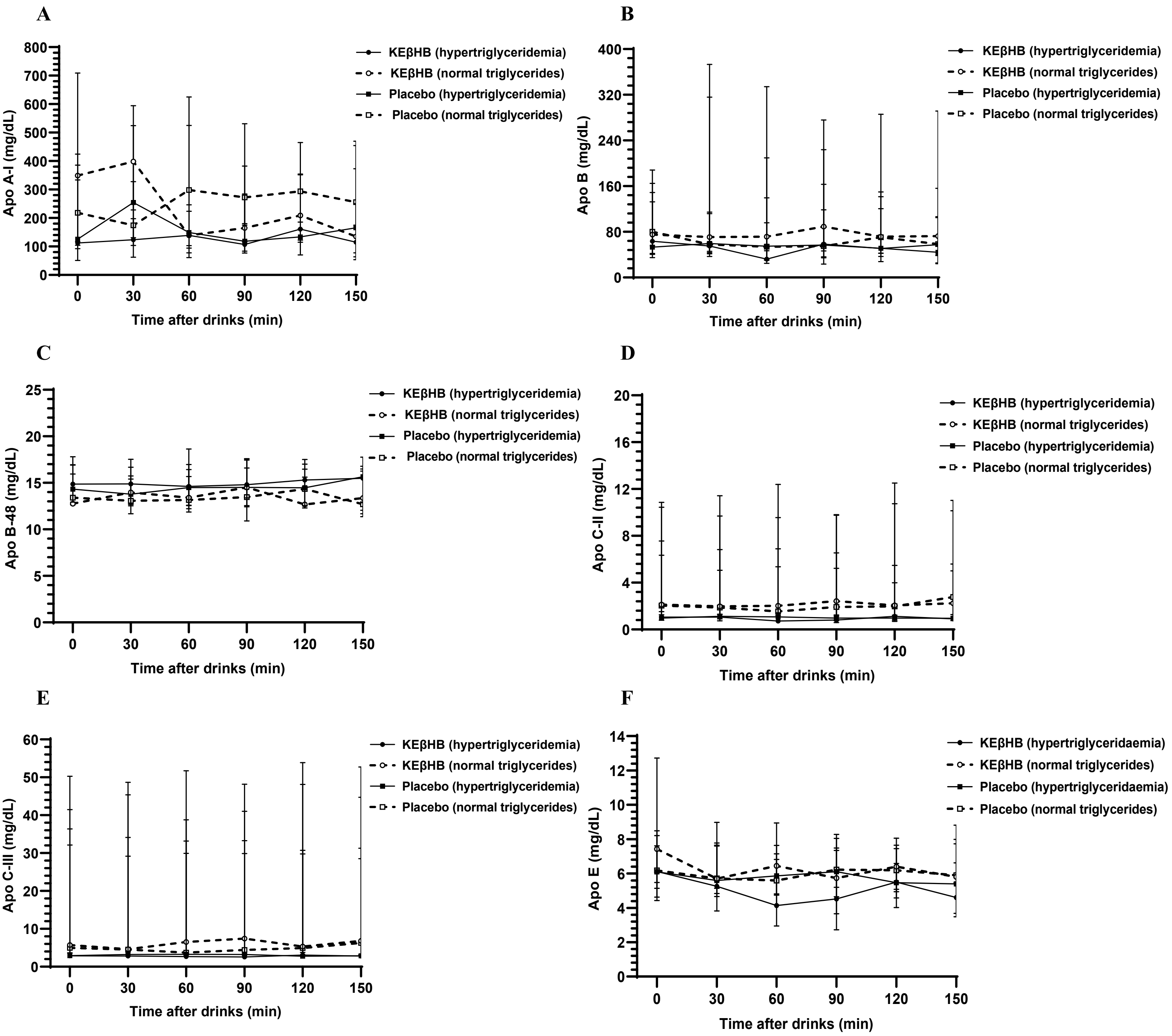

Supplementary figure 1. *Changes in plasma concentrations of apolipoproteins after the KEβHB versus placebo drinks according to participant's circulating triglyceride levels at baseline.*

Footnotes: Participants were stratified according to the median values of their circulating triglyceride levels at baseline. Data were presented as median and interquartile range at 0 (fasted), 30, 60, 90, 120, and 150 minutes in panels A – F.

Abbreviations: Apo, apolipoprotein; AUC, area under the curve; KEβHB, ketone monoester (β-hydroxybutyrate).

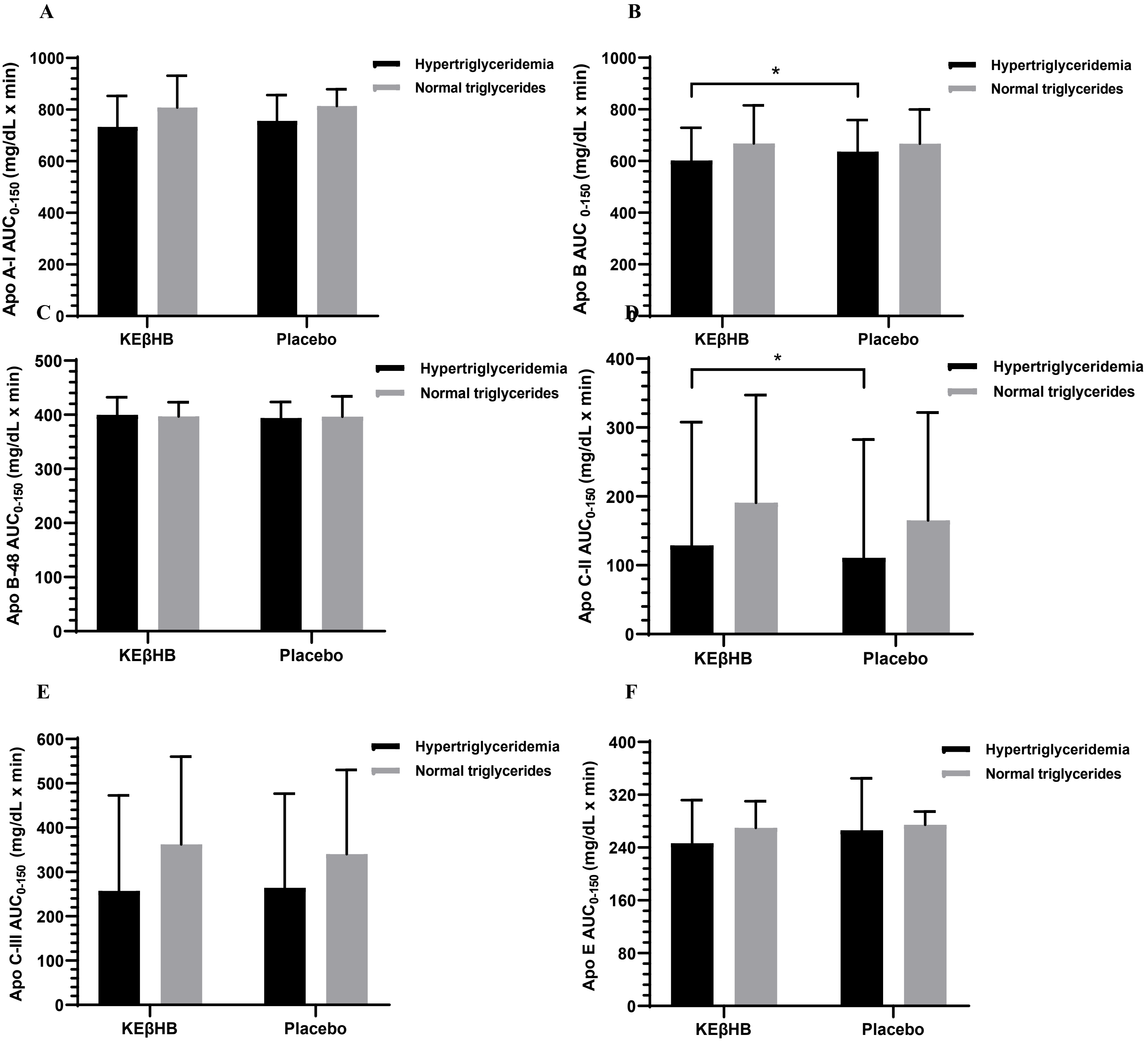

Supplementary figure 2. *Changes in plasma concentrations of apolipoproteins after the KEβHB versus placebo drinks according to participant's circulating triglyceride levels at baseline.*

*Footnotes: Participants were stratified according to the median values of their circulating triglyceride levels at baseline. Total area under the curve (AUC_{0-150}) after both the KEβHB and placebo drinks are presented as mean ± standard error of mean in panels A – F. * $P < 0.05$ for the difference between the KEβHB and placebo drinks.*

Abbreviations: Apo, apolipoprotein; AUC, area under the curve; KEβHB, ketone monoester (β-hydroxybutyrate).

Supplementary table 1. Overall effect of the KEβHB versus placebo on apolipoproteins, stratified by circulating levels of triglycerides at baseline

Apolipoproteins [1]	Group/significance [2]	Fasting serum triglycerides	
		Hypertriglyceridemia	Normal triglycerides
Apo A-I (mg/dL × min)	KEβHB (n=18)	732.54 ± 40.04	808.19 ± 40.92
	Placebo (n=18)	756.46 ± 33.23	814.17 ± 21.60
	Δ (KEβHB-placebo)	-23.91 (-77.60, 29.77)	-5.98 (-69.81, 57.86)
	d	0.22	0.06
	p value	0.334	0.834
Apo B (mg/dL × min)	KEβHB (n=18)	601.88 ± 42.18	667.70 ± 49.19
	Placebo (n=18)	636.47 ± 40.64	667.38 ± 44.04
	Δ (KEβHB-placebo)	-34.59 (-68.52, -0.66)	0.32 (-57.02, 57.67)
	d	0.28	<0.01
	p value	0.047*	0.990
Apo B-48 (mg/dL × min)	KEβHB (n=18)	399.68 ± 10.93	396.79 ± 8.76
	Placebo (n=18)	393.84 ± 9.95	396.66 ± 12.47
	Δ (KEβHB-placebo)	5.83 (-4.18, 15.84)	0.13 (-20.93, 21.20)
	d	0.19	<0.01
	p value	0.216	0.989
Apo C-II (mg/dL × min)	KEβHB (n=18)	128.95 ± 59.58	190.81 ± 52.14
	Placebo (n=18)	111.95 ± 57.13	165.21 ± 52.22
	Δ (KEβHB-placebo)	18.0 (2.12, 33.87)	25.60 (-12.58, 63.78)
	d	0.10	0.16
	p value	0.031*	0.161
Apo C-III (mg/dL × min)	KEβHB (n=18)	257.16 ± 71.95	362.28 ± 65.97
	Placebo (n=18)	264.47 ± 70.74	340.19 ± 63.31
	Δ (KEβHB-placebo)	-7.31 (-17.19, 2.56)	22.09 (-40.13, 84.3)
	d	0.03	0.11
	p value	0.126	0.437
Apo E (mg/dL × min)	KEβHB (n=18)	246.48 ± 21.84	269.80 ± 13.48
	Placebo (n=18)	266.32 ± 26.20	274.41 ± 6.70
	Δ (KEβHB-placebo)	-19.84 (-42.42, 2.73)	-4.61 (-37.36, 28.13)
	d	0.27	0.14
	p value	0.077	0.754

Footnotes: [1] AUCs were the total AUCs of log-transformed variables that were calculated from 0 to 150 minutes. Values are presented as mean ± standard error of mean (SEM), and their changes are presented as mean (95% confidence interval).

[2] P values were obtained from paired t-test, and variables were log-transformed for the paired t-test.

Abbreviations: Apo, apolipoprotein; AUC, area under the curve; KEβHB, ketone monoester (β hydroxybutyrate).
